# Probing thermal transport across amorphous region embedded in a single crystalline silicon nanowire

**DOI:** 10.1038/s41598-020-57514-9

**Published:** 2020-01-21

**Authors:** Yunshan Zhao, Xiangjun Liu, Ashutosh Rath, Jing Wu, Baowen Li, WuXing Zhou, Guofeng Xie, Gang Zhang, John T. L. Thong

**Affiliations:** 1 Department of Electrical and Computer Engineering, National University of Singapore, Singapore, 117583 Republic of Singapore; 2 Institute of Micro/Nano Electromechanical System, College of Mechanical Engineering, Donghua University, Shanghai, 201620 China; 3 Department of Materials Science and Engineering, National University of Singapore, Singapore, 117575 Republic of Singapore; 4 Institute of Materials Research and Engineering, Agency for Science, Technology and Research, Singapore, 138634 Republic of Singapore; 5 Department of Mechanical Engineering, University of Colorado, Boulder, 80309 USA; 6 School of Materials Science and Engineering, Hunan University of Science and Technology, Xiangtan, 411201 China; 7 Institute of High Performance Computing, Singapore, Singapore 138632 Republic of Singapore

**Keywords:** Nanowires, Nanowires

## Abstract

While numerous studies have been carried out to characterize heat transport behaviours in various crystalline silicon nanostructures, the corresponding characteristics of amorphous one-dimension system have not been well understood. In this study, we amorphize crystalline silicon by means of helium-ion irradiation, enabling the formation of a completely amorphous region of well-defined length along a single silicon nanowire. Heat conduction across both amorphous region and its crystalline/amorphous interface is characterized by an electron beam heating technique with high measurement spatial resolution. The measured thermal conductivity of the amorphous silicon nanowire appears length-independence with length ranging from ~30 nm to few hundreds nm, revealing the fully diffusons governed heat conduction. Moreover, unlike the size-dependent interfacial thermal conductance at the interface between two one-dimensional crystalline materials, here for the first time, we observe that the interface thermal conductance across the amorphous/crystalline silicon interface is nearly independent of the length of the amorphous region. This unusual independence is further supported by molecular dynamics (MD) simulation in our work. Our results provide experimental and theoretical insight into the nature of interaction between heat carriers in crystalline and amorphous nano-structures and shed new light to design innovative silicon nanowire based devices.

## Introduction

While crystalline materials are commonly studied, studying the thermal properties of non-crystalline materials, including all kinds of inorganic, organic, biological materials and their hybrid structures, is equally important, as it can significantly enrich our knowledge of energy transport in non-periodic lattices. Moreover, emergent technologies, like new generations of wearable electronics and soft machines, often exploit non-crystalline materials^1–3^. In such materials, the phonon picture fails for atoms arranged in a disordered fashion, where heat is carried in a random channel among series of independent oscillators^4–6^. This uncorrelated oscillator picture could be attributed to Einstein, who in 1911^7^ predicted the low thermal conductivity limit for a completely amorphous disordered material, namely the “amorphous limit”, in which the interactions between random oscillators lead to heat conduction. Since then, this amorphous limit model has been debated in numerous studies, since the values of thermal conductivity of amorphous solids reported were much higher. For amorphous silicon, a ubiquitous component of modern electronic devices, the thermal conductivity based on the calculated amorphous limit is below 2 W/m·K^8^. Recent studies have shown that the thermal conductivity of amorphous silicon could be much higher^9,10^ and as high as 4–5 W/m·K has been claimed^11^. The fact of much higher thermal conductivity than the amorphous limit prediction for amorphous silicon has spurred numerous theoretical calculations to understand how heat carriers transport in amorphous silicon and what their individual contributions are towards the overall thermal conductivity.

According to Allen-Feldman theory^12,13^, although diffusons (delocalized and non-propagating) have short mean free paths (less than 10 nm), they dominate in thermal transport in amorphous silicon due to their large proportion. The propagating propagons (delocalized and propagating) are believed to contribute as well, considering their relatively large mean free paths. The other carriers are locons, which are localized and non-propagating, thus contributing negligibly to the thermal transport^14,15^. Theoretically, by means of molecular and lattice dynamics simulation, He *et al*.^16^ predicted that the propagating vibrations would contribute half of the thermal conductivity of amorphous silicon, despite its small proportion of around 3%; they also showed that their long mean free path saturated around 1 µm. An even longer mean free path of propagons was found by Kwon *et al*.^11^ and these propagons would account for 30% of the thermal conductivity of bulk amorphous silicon. A dominant contribution of propagating elastic waves was presented by Moon *et al*.^17^ and these propagating modes are significantly scattered by fluctuations of elastic modulus instead of anharmonicity.

To experimentally observe different contributions of heat carriers in thermal transport of amorphous silicon, fabrication techniques that are capable to generate high-level amorphous state with well-controlled dimensions are necessary. Typically, there are two kinds of popular sample fabrication techniques – sputtering^8,18,19^ and chemical vapour deposition^10,20,21^. It is noticed that all the previously reported amorphous silicon nanostructures are either in thin-films or bulk forms, in which the inhomogeneities and voids could never be fully avoided^22–24^ and these unavoidable factors would influence the thermal properties significantly by providing extra transport channels for heat carriers. As for amorphous silicon nanowires, despite their promising applications in lithium-ion batteries^25^ and photovoltaics^26^, their thermal transport property has not been deeply explored.

In this work, we amorphize selective regions of a single-crystalline silicon nanowire with well-defined lengths by using helium-ion irradiation. By means of an electron beam heating technique, the thermal conductivity of the amorphous regions of various lengths is probed, showing a constant value around 1.9 ± 0.25 W/m·K. This fully diffusons governed heat conduction of amorphous silicon nanowires neglects any contributions from the propagons considering their long mean free paths. Interestingly, we show that amorphous/crystalline interface thermal conductance is independent of the length of the amorphous region and this independence differs from that of other crystalline/crystalline interfaces. Further molecular dynamics (MD) calculation and theoretical model support the experimental observation, where length-independent density of states (DOS) of amorphous silicon nanowire is demonstrated.

## Results and Discussions

### Creation of amorphous segments along silicon nanowire

**Figure 1:**
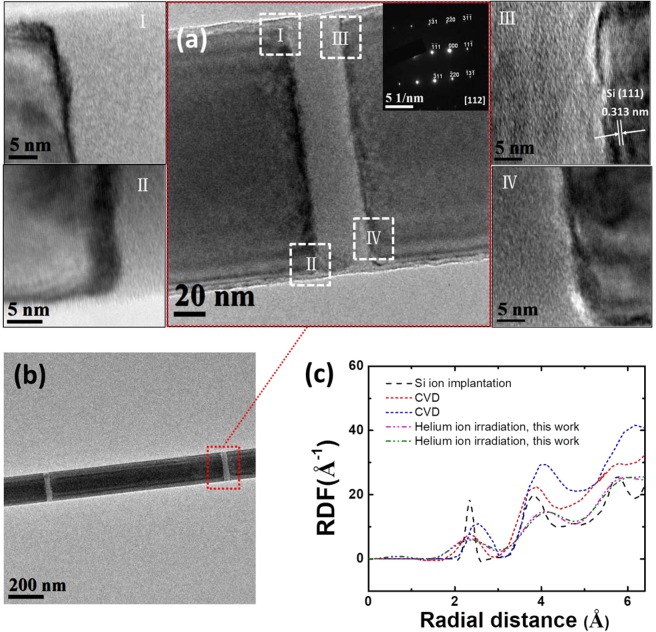
Characterization of crystalline/amorphous interface (CAI) along one single silicon nanowire. (**a**) Bright field Transmission Electron Microscopy (TEM) image of calibrated silicon nanowire. High-resolution TEM (HRTEM) image of the square marked regions in (**a**) was shown in Figures I, II, III and IV and selected area electron diffraction (SAED) pattern on the crystalline region was shown in the inset of Fig. 1a. The lattice spacing 0.313 nm corresponds to (111) direction^30^. The scale bar was illustrated in each figure. **(b**) One single silicon nanowire with two amorphous regions embedded along it with a spacing ~1 µm. The red square region is highlighted in in. (**a,c)** Radial distribution function (RDF) of amorphous silicon. The RDF of amorphous silicon nanowire fabricated in our current work, and compared with those fabricated by silicon ion implantation^32^ and chemical vapor deposition (CVD)^11^.

We had previously demonstrated the creation of an amorphous region of desired length along a single-crystal silicon nanowire by helium ion irradiation^27^. To obtain an amorphous silicon region with a much cleaner and sharper interface, we modified the irradiation conditions by calibrating the doses before irradiating the target nanowires, with details described in Supporting Information Fig. S1. A dose of 1.3 × 10^17^ cm^−2^ was chosen as the final irradiation dose, sufficient to amorphize the silicon without introducing significant roughness at the crystalline/amorphous interface (CAI) and most importantly, without causing any appearance of voids. Using this approach, we created amorphous segments of varying lengths in 15 such nanowires, as shown in Supporting Information Fig. S2. To ensure that every silicon atom has equal probability of being impinged by a helium ion, the ion beam scan step size is kept to ~0.25 nm, smaller than the lattice spacing of silicon, considering the growth direction along the [111] direction for the silicon nanowires used in this study. Figure 1(a) shows a short amorphous region with length of ~30 nm created along one single nanowire without noticeably affecting the crystallinity of the adjacent unirradiated regions, as evidenced by the single-crystalline diffraction pattern in the inset as well as the high resolution transmission electron microscope (HRTEM) image (acquired using a JEOL 2010F TEM, at 200 kV) shown in Fig. 1 (I)-(IV). It was reported by Liang *et al*. that in the crystalline and amorphous sandwiched structures, phonon interference effect would exist if the thickness of the central amorphous silicon film were less than 5 nm^28^. A similar scale of phonon penetration depth was also observed in amorphous silicon dioxide^29^. To avoid the possible phonon interference effect, in this study, the minimum length of amorphous silicon section is kept much longer than the phonon penetration depth. The low magnification image of one silicon nanowire is shown in Fig. 1(b), where two amorphous regions irradiated by two different doses (1.3 × 10^17^ cm^−2^ and 1.2 × 10^17^ cm^−2^) are created in the process of dose calibration. From the HRTEM image, the interface roughness is of the order of a few nanometers, and this abrupt CAI would be used for the interface thermal conductance measurement discussed later. The measured lattice spacing is 0.313 nm as marked in Fig. 1 (III), corresponding to the (111) crystalline direction^30^.

To show the degree of atomic disorder in helium ion irradiated silicon nanowires, the radial distribution function (RDF) is extracted from the selected area electron diffraction (SAED) pattern of amorphous regions, using RDFTools^31^ software package for the Gatan Digital Micrograph software. As shown in Fig. 1(c), the positions of peaks in amorphous silicon fabricated by different approaches (i.e., CVD^11^, silicon ion implantation^32^ and helium ion irradiation in this work) overlap with each other, while the peak width differs a lot. Typically, the width of the RDF peak contains information for the dispersion of the respective interatomic distances, and its integral intensity corresponds to the number of atomic pairs. For amorphous silicon, the position of the first peak in the RDF corresponds to the first-nearest-neighbor distance (i.e., the mean bond-length) and the width corresponds to variations in the bond-lengths. Thus, RDF data suggests our samples exhibit relatively high degree as the RDF peak observed in our samples is broad in width and small in magnitude. We also show the difference in damage incurred in amorphous silicon between silicon ion implantation and helium ion irradiation by performing Monte Carlo simulation based on a binary collision approach (TRIM/SRIM) and the result is discussed in Supporting Information Fig. S3. For a typical heavy ion, each collision cascade is most likely to produce divacancies and more complicated defect structures, where a substantial cluster containing possible voids and impurities easily comes into being^33^. Thus, it is difficult to create well-controlled point defects and hence completely amorphous disorder in silicon by heavy ions; for a light ion, it creates point and point-like defects rather than large damage clusters, due to the relatively uniform spatial defect profile^27^. Thus dense amorphous silicon without detectable voids can be obtained by light ion irradiation, which possibly accounts for the difference in RDF of amorphous silicon obtained by silicon ion implantation and helium ion irradiation in Fig. 1(c).

### Thermal conductivity measurement

**Figure 2:**
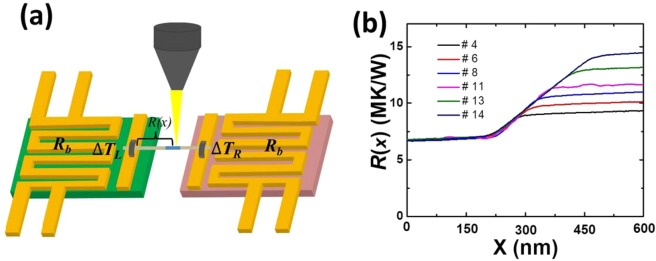
Measurement of electron beam heating technique. (**a**) Sketch of electron beam heating technique. *∆T*_*L*_ and *∆T*_*R*_ is temperature rise in left and right Pt thermometer, separately. *R(x)* is the cumulative thermal resistance along the suspended nanowire from the left thermometer to the scanning point of *x*. The yellow pyramid denotes the irradiating electron beam. (**b**) Length-dependent cumulative thermal resistance of different nanowires, which are #4, #6, #8, #11, #13 and #14. The slope of amorphous regions overlaps with each other, even for different nanowires with different amorphous lengths, meaning that the amorphous regions with various lengths keep constant thermal conductivity around 1.9 ± 0.25 W/m·K. The cumulative thermal resistance of the rest silicon nanowires is shown in Supporting Information Fig. S5.

The thermal conductance along the nanowires with amorphous segments was measured using an electron beam heating technique that we had previously developed, with details discussed elsewhere^27,34–36^. The measured Silicon nanowires with diameter ~160 nm were purchased from Sigma-Aldrich 730866) with a [111] growth direction. As shown in the sketch in Fig. 2(a), a SEM electron beam is used to heat up the suspended nanowire on a Micro-Electro-Thermal System (METS) device and the temperature rise at two ends of nanowire is probed by the two platinum (Pt) loops (thermometers). *∆T*_*L*_ and *∆T*_*R*_ denote the temperature rise in the left and right Pt thermometers, respectively. At a thermal steady state, the cumulative thermal resistance *R*(*x*) at the position of *x* along the measured nanowire is extracted as

1 $$R(x)={R}_{b}\{\frac{{\alpha }_{0}-\alpha (x)}{1+\alpha (x)}\}$$

where *R*_*b*_ is the thermal resistance of suspending SiN_x_ beams, *α*_0_ is the temperature rise ratio of *∆T*_*L*_*/∆T*_*R*_ when a DC current is introduced to the left thermometer, and *α*(*x*) is for the ratio of *∆T*_*L*_(*x*)*/∆T*_*R*_(*x*) when a spot *x* along the nanowire is irradiated and heated up by the electron beam without the DC current input. The measurement of *α*_0_ could give information about the thermal resistance of the measured nanowires, since *R*_*T*_ = (*α*_0_-1)·*R*_*b*_, where *R*_*T*_ denotes the total thermal resistance of measured nanowire, including its contact thermal resistance with the left & right thermometer islands. To record the thermal resistance change of silicon nanowire, *α*_0_ was measured for each METS device before and after helium ion irradiation and the change of *α*_0_ is shown in Supporting Information Fig. S4 and Note 1. The increase ratio of *α*_0_ keeps linearly increasing with the increasing length of amorphous segments, indicating the gradual increasing in the thermal resistance of measured silicon nanowire, considering constant *R*_*b*_ for each METS device and constant contact thermal resistance once the METS devices are fabricated. The final thermal conductivity is obtained by *κ* = 1/(*dR(x)*/*dx*)*/A*, where *A* is the cross-sectional area of silicon nanowire, determined from the TEM image.

The thermal resistance of six silicon nanowires measured across the amorphous segments is presented in Fig. 2(b), for Samples #4, #6, #8, #11, #13 and #14. Although silicon nanowires have different amorphous region lengths (*L*) on different METS devices, the thermal resistance curves of the (unirradiated) crystalline parts of the silicon nanowires are either overlapping or parallel to each other, implying the same thermal conductivity, which is 50.2 ± 2.3 W/m·K, obtained by linearly fitting the curves of crystalline regions and taking average of all the measured samples. This length-independence of thermal conductivity is captured by the e-beam heating technique, considering the minimal length change in the crystalline silicon part. For the (irradiated) amorphized region, the thermal resistance curves of different silicon nanowires overlap each other, implying a constant thermal conductivity, which is calculated to be 1.9 ± 0.25 W/m·K and corresponds with data in references^10,19,27^. The errors here were due to the nanowire dimension characterization, linearly fitting cumulative thermal resistance (*R(x)*) curve as well as the METS device calibrations (*R*_*b*_ and temperature coefficient of resistance (TCR)). Different from the previous dimension changing treatment^37^, the helium ion irradiation can locally engineer the thermal conductivity of one single silicon nanowire without affecting the surface morphology of its crystalline section, which provides a handle on the thermal design of silicon-nanowire-based devices. It should be noted that for all the silicon nanowires in different METS devices, the measured values of the thermal conductivity of both crystalline and amorphous silicon regions are repeatable, indicating that the helium ion irradiation process is consistent and that the experimental measurement can be carried out with high reproducibility. The cumulative thermal resistance curves of the remaining silicon nanowires are summarized in Supporting Information Fig. S5.

**Figure 3:**
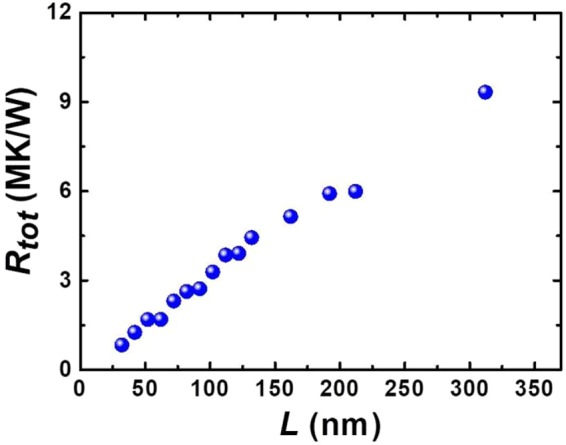
Thermal transport in amorphous silicon nanowires. The increased total thermal resistance (*R*_*tot*_) across the amorphous regions with different lengths. The linear dependence of cumulative thermal resistance on amorphous region lengths is shown and the thermal conductivity of amorphous silicon can be obtained by linearly fitting the curve, which is 1.7 ± 0.1 W/m·K, similar to that obtained from linearly fitting the cumulative thermal resistance previously in Fig. 2(b).

To better understand heat carrier transport in amorphous silicon at short length scales, we extract the total thermal resistance across the entire amorphous segment with different lengths (*L*) and summarize the data in Fig. 3. The thermal resistance is seen to increase linearly with the length of the amorphous segment, and this linear relationship is observed for all the measured samples. Similarly, we calculate the thermal conductivity of the amorphous silicon segments using the formula *κ = *1/(*dR(x)*/*dx*)*/A*, which is around 1.7 ± 0.1 W/m·K, and is the same as that obtained by a linear fit of the cumulative thermal resistance obtained previously in Fig. 2(b). The consistency of the value of thermal conductivity obtained by these two approaches doubly confirms the repeatability in extracting the final thermal conductivity. For the amorphous silicon nanowires studied in this work, the contribution of propagons to thermal conductivity is negligible considering the diameter filtering effect and their long mean free paths^11,16^, and the length independent thermal conductivity is therefore observed.

### Thermal transport across crystalline/amorphous interface (CAI)

**Figure 4:**
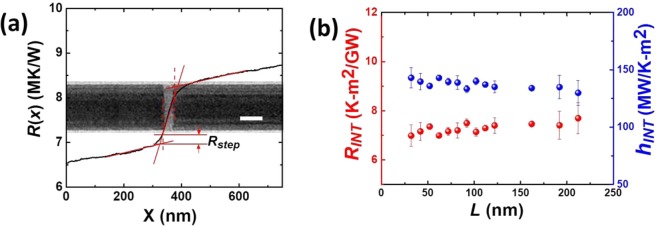
Measurement of interfacial thermal resistance of CAI. (**a**) Cumulative thermal resistance across the CAI in Sample #1. *R*_*step*_ denotes the thermal resistance jump across CAI, obtaining from linear fitting *R*(x) at both crystalline and amorphous regions, elongating across the interface and thus taking the step. *R*_*step*_ is used to calculate the interface thermal conductance of CAI. The scale bar is 75 nm. (**b**) Interface thermal conductance (*h*_*INT*_) and interface thermal resistance (*R*_*INT*_) of Sample #1-Sample #14. *h*_*INT*_ = 1/(*R*_*step*_·*A*), where *A* is the cross section area of each silicon nanowire and *R*_*INT*_ = 1/*h*_*INT*_.

As mentioned earlier, the heat carriers in amorphous solids like amorphous silicon can be classified into three types of vibration: progagons, diffusons, and locons, and only progagons show a propagating behaviour. How these heat carriers interact with the phonons across one crystalline/amorphous interface remains a subject of debate and there appears to be no advanced approaches that have been developed to account for how these heat carriers interact to transfer energy across an interface^38^. Interestingly, the creation of CAIs along one single silicon nanowire in our study provides an excellent platform to study the interface thermal transport given the clean crystalline/amorphous interface obtained by helium ion irradiation. For the electron beam heating measurement, when the electron beam traverses the interface, there is a step change in the cumulative thermal resistance as marked by the arrow in Fig. 4(a), which denotes intuitively the interface thermal resistance (ITR). To determine the ITR, we linearly fit *R*(*x*) in both crystalline and amorphous portions that are far away from the interface and extrapolate these two fitted lines to intersect at the interface. The step change in thermal resistance at the two intersects is defined as ITR, which is *R*_*step*_. Fig. 4(a) shows the detailed fitting process by using Sample #1 and the inset is the CAI clearly seen in the TEM image. For further calculation of interface thermal conductance, the average ITR is employed, taking the average of *R*_*step*_ of two crystalline/amorphous interfaces across CAI and the interface thermal conductance (*h*_*INT*_) is defined as *h*_*INT*_ = 1/(*R*_*step*_·*A*), where *A* is the cross sectional area of each silicon nanowire.

Similarly, ITR for the remaining silicon nanowires measured are extracted by this method and the data is summarized in Fig. 4(b). Interestingly, *h*_*INT*_ is nearly independent of the length of the amorphous segments, which differs from other solid interface systems where *h*_*INT*_ depends strongly on the length of each portion and we will discuss this unusual independence in the next section. The interface thermal resistance, *R*_*INT*_, is shown as well in Fig. 4(b), which is defined as *R*_*INT*_ = 1/*h*_*INT*_. Our measured ITR between amorphous and crystalline silicon shows same order of magnitude in value to that predicated by theoretical calculation^38^.

### Molecular dynamics simulation and theoretical analysis

**Figure 5:**
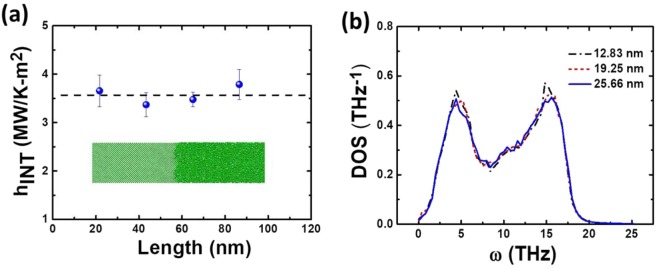
Molecular dynamics (MD) simulation. (**a**) The dependence of interface thermal conductance on the length of amorphous segment from MD calculation. The detailed heat flux and temperature jump across the interface are shown in Supporting Information Fig. S6. The inset is crystalline/amorphous structure. **(b)** The calculated density of states (DOS) for amorphous silicon nanowires with various length. The DOS is nearly independent on the length of amorphous silicon nanowire.

Molecular dynamics (MD) simulations are employed to study the thermal transport across the CAI using the LAMMPS package. The calculation details are provided in Supporting Information Fig. S6 and Supporting Information Note 2, and the structure is shown in Supporting Information Fig. S7. For comparison, we also study the interface thermal conductance (ITC) between two crystalline SiNWs, by artificially increasing the atomic mass of one section from 28 (for silicon) to 72, which is shown in Supporting Information Fig. S6(a,c). It is clearly observed that ITC increases monotonically with the increase of length, similar to those observed previously^38^, which turns out to be a general feature for interfacial thermal transport. With the length of crystalline material increasing, more phonon modes are excited and these excited phonons would make more contributions to interfacial thermal transport. Nevertheless, at the interface between crystalline and amorphous Si NWs, the length of amorphous section has little effect on ITC, giving rise to a nearly constant value within the considered range of lengths, as shown in Fig. 5(a), which is consistent with the length independent ITC observed at graphene-water interface^39^ and graphene-polymer interface^40^. In all the simulated domains, the length of crystalline segment is kept constant (21.7 nm) and the length of amorphous segment changes from 21.7 nm to 86.9 nm.

To better understand the abnormal independence of ITC on the length of amorphous silicon, we recall the theoretical model developed by Correa *et al*.^41^, which is widely employed to calculate the phonon transmission rate between the two contacts and puts as,

2 $${\rm{\tau }}(\omega )=\frac{\pi }{2}(\frac{{D}_{1}(\omega )}{{m}_{1}^{2}}+\frac{{D}_{2}(\omega )}{{m}_{2}^{2}})\frac{{K}^{2}}{{\omega }^{2}}$$

where *m*_1_ (*m*_2_) are the average weights of atoms in the two contacts, *D*_1_(*ω*) (*D*_2_(*ω*)) is their phonon density of states (PDOS), *K* is the effective coupling spring constant describing the interaction strength between the two contacts, and *ω* is phonon frequency. In contrast to the continuous PDOS of bulk silicon, MD simulations clearly show that there are many discrete peaks in PDOS spectra of crystalline Si NW^42^. Because the shortest phonon wavelength is twice the lattice constant, the PDOS in high energy regime (short wavelength) is not sensitive to the wire length. However, for low frequency phonons, there is obvious length dependence^42^. For a short Si NW, the low energy phonon density is very small, and with the increase of length, more and more phonons are excited, which results in the increase of ITC. However, in amorphous Si NW and other disorder solids, only acoustic-like vibrational excitations exist, with each excitation consisting of several different normal modes with different frequencies, which is in contrast to the case where single acoustic excitation has single normal mode in the crystal^43,44^. As a result, the acoustic-like excitation in amorphous materials attenuates rapidly, is strongly localized, and cannot be described as a plane wave. Moreover, strong scattering and breakdown of the Debye continuum approximation have been observed in the range of low frequency^45^. Therefore, the DOS of amorphous Si NW is typically believed to be insensitive to its transport length. To verify this, MD simulation was performed to show the length independent DOS for amorphous Si NW and the result is shown in Fig. 5(b). With increasing length of amorphous silicon nanowires, the DOS in low frequency regimes (for acoustic-like vibrational excitations) remains nearly unchanged. This length-insensitive DOS accounts for the length independent ITC observed both experimentally and theoretically. The detailed DOS calculation is shown in Supporting Information Fig. S8 and Note 3.

## Conclusions

In summary, we experimentally probed the thermal transport in amorphous silicon nanowire with segment length down to ~30 nm for the first time. By means of helium ion irradiation, one single crystalline silicon nanowire was amorphized into completely disordered amorphous regions with well-controlled lengths, making the measurement of length-dependent thermal conductivity in amorphous silicon nanowire possible. An electron beam heating technique was employed to measure the thermal transport across the amorphous segments along one silicon nanowire. The interface thermal transport across crystalline/amorphous interface was measured as well, and it was shown that this is independent of the length of the amorphous segments. This atypical independence in our study differs from other solid interface systems, where the strongly correlated dependence of interface thermal conductance on the system size was observed. All the experimental observations were further supported by theoretical calculations in our study.

## Supplementary information

Probing thermal transport across amorphous region embedded in a single crystalline silicon nanowire.
