# Supplementary material for: Probing thermal transport across amorphous region embedded in a single crystalline silicon nanowire
Source: Sci Rep. 2020 Jan 21;10:821. doi: 10.1038/s41598-020-57514-9 (PMC6972709; doi:10.1038/s41598-020-57514-9)
Supplement: Supplementary file 1 — Probing thermal transport across amorphous region embedded in a single crystalline silicon nanowire. [file 41598_2020_57514_MOESM1_ESM.docx]

Supporting Information

**Probing thermal transport across amorphous region embedded in a single crystalline silicon nanowire**

Yunshan Zhao^1^,*, Xiangjun Liu^2^, Ashutosh Rath^3^, Jing Wu^4^, Baowen Li^5^, WuXing Zhou^6^, Guofeng Xie^6^, Gang Zhang^7^,*, John T L Thong^1^,*

^1^Department of Electrical and Computer Engineering, National University of Singapore, Singapore 117583, Republic of Singapore

^2^Institute of Micro/Nano Electromechanical System, College of Mechanical Engineering, Donghua University, Shanghai, 201620, China

^3^Department of Materials Science and Engineering, National University of Singapore, Singapore 117575, Republic of Singapore

^4^Institute of Materials Research and Engineering, Agency for Science, Technology and Research, Singapore 138634, Republic of Singapore

^5^Department of Mechanical Engineering, University of Colorado, Boulder 80309, USA

^6^School of Materials Science and Engineering, Hunan University of Science and Technology, Xiangtan 411201, China

^7^Institute of High Performance Computing, Singapore, Singapore 138632, Republic of Singapore


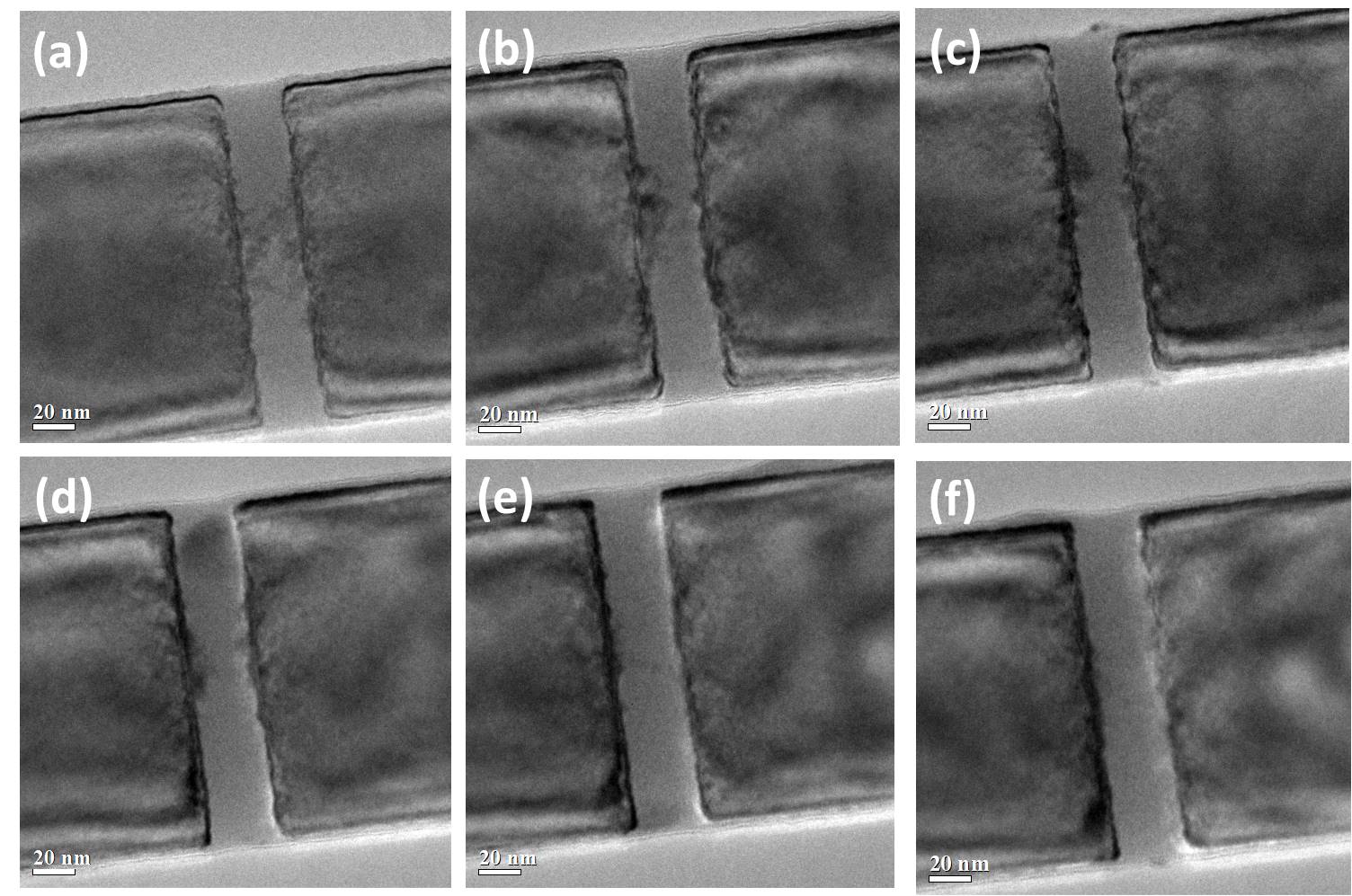


**Supplementary Figure 1 | TEM characterization across amorphous regions along one single crystalline silicon nanowire.** (a)-(f) are TEM images of amorphous regions (length ~32 nm) under dose of 8×10^16^, 9×10^16^, 1×10^17^, 1.1×10^17^, 1.2×10^17^ and 1.3×10^17^ cm^-2^, respectively. The crystalline tails become smaller with increasing the dose and the dose of 1.3×10^17^ cm^-2^ is chosen as the final irradiation dose in this study. The scale bars are shown in each figure.


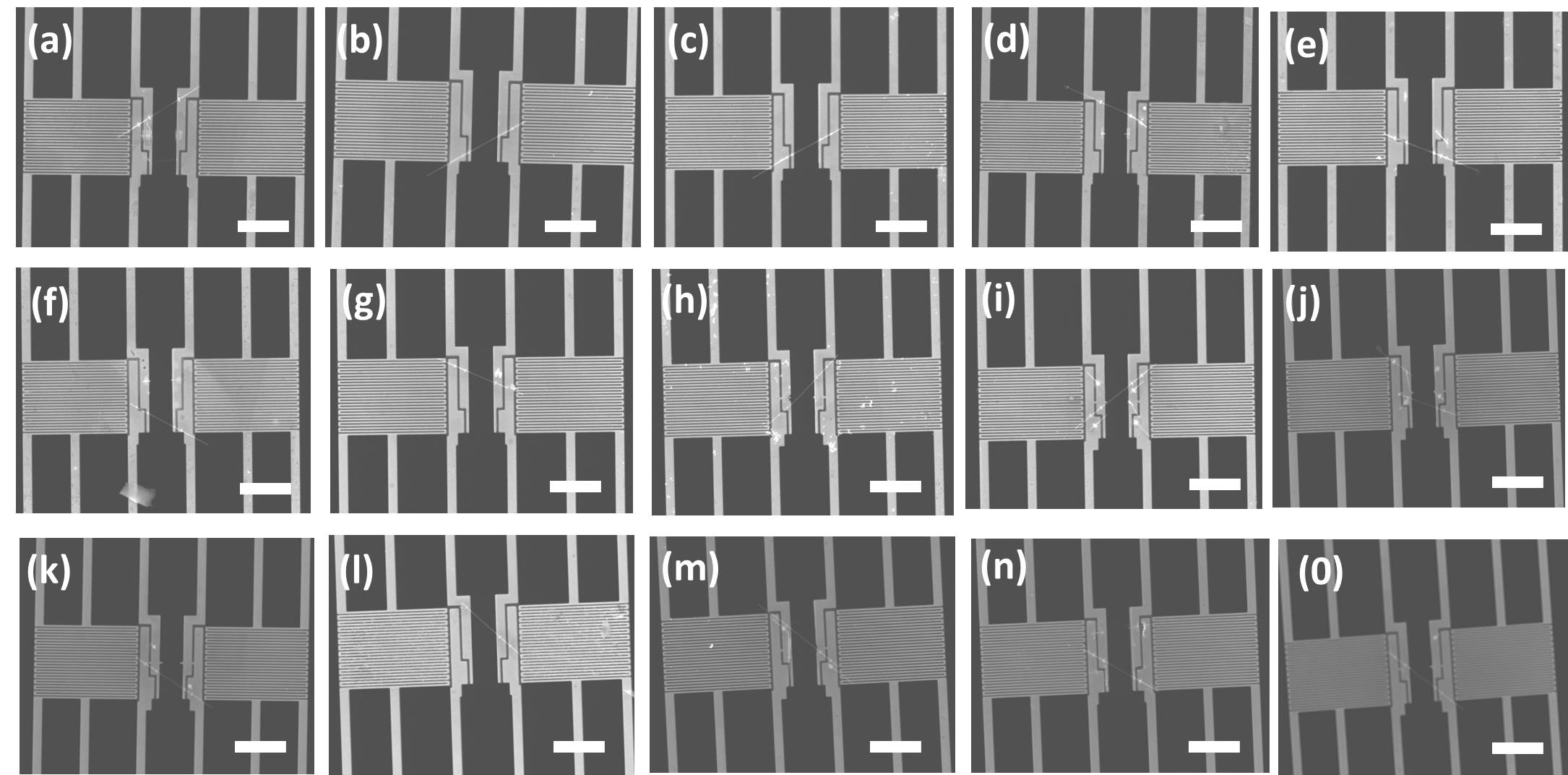


**Supplementary Figure 2 | Silicon nanowires on METS device.** (a)-(o) are SEM images of measured samples #1 to #15, respectively. The scale bars are 10 μm.


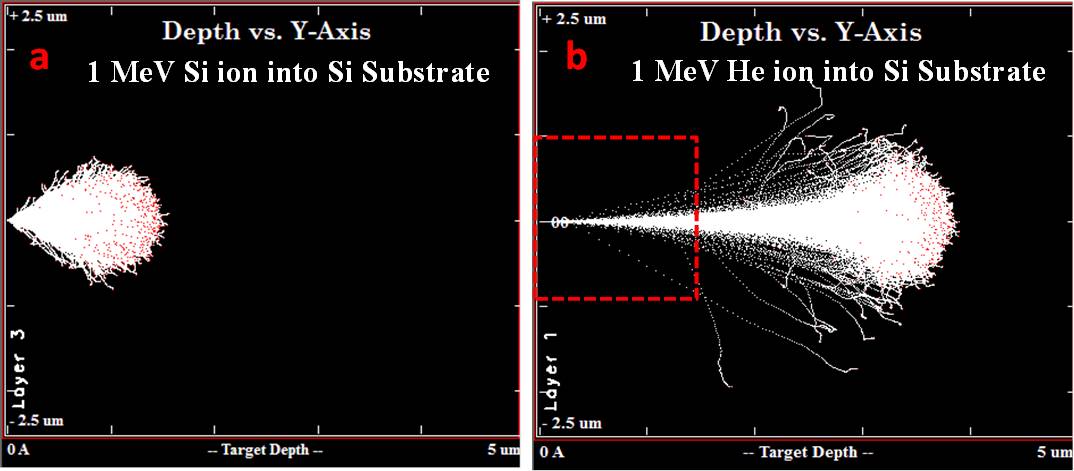


**Supplementary Figure 3 | SRIM simulation for 9999 silicon ions (a) and 9999 helium ions (b) implanted into silicon substrate with width of 5 µm.** The energy for these two ions is same as 1 MeV. The red dashed square in (b) has the same depth with Si ion maximum projected length in (a).


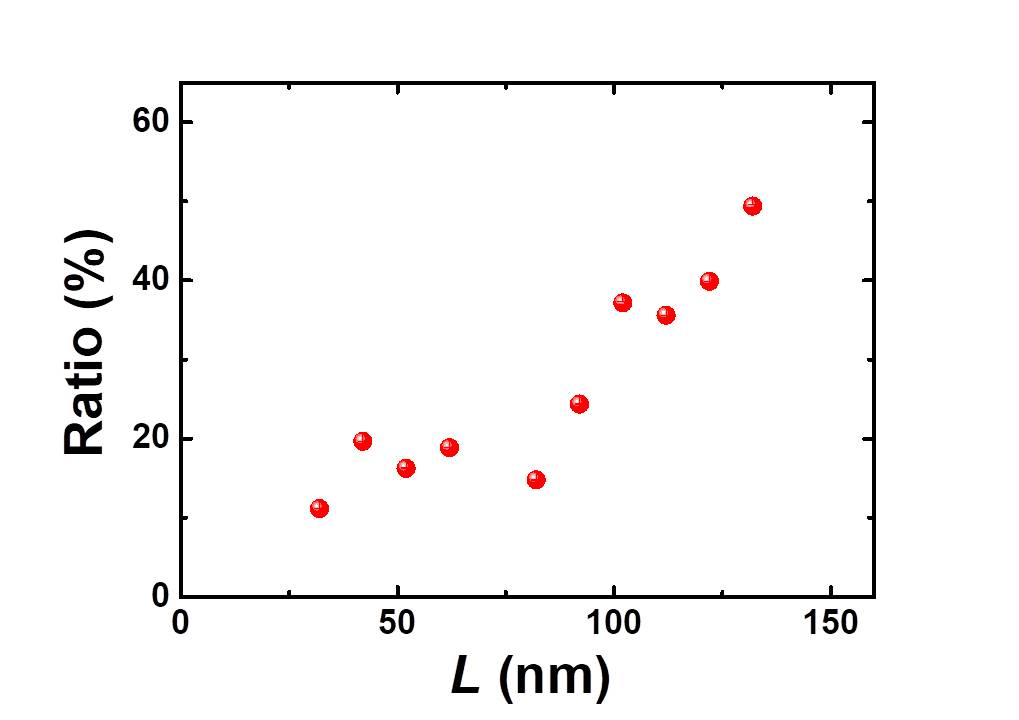


**Supplementary Figure 4 | Increase ratio of thermal resistance of measured silicon nanowires.** Ratio = (*R_T_,* *_irradiation_* - *R_T,non-irradiation_*)/*R_T,non-irradiation_*, which is (*a_0_,* *_irradiation_*-*a_0_,* *_non-irradiation_*)/ *a_0_,* *_non-irradiation_*, considering constant *R_b_* and contact thermal resistance between nanowires and left (right) Pt loops before and after helium ion irradiation.


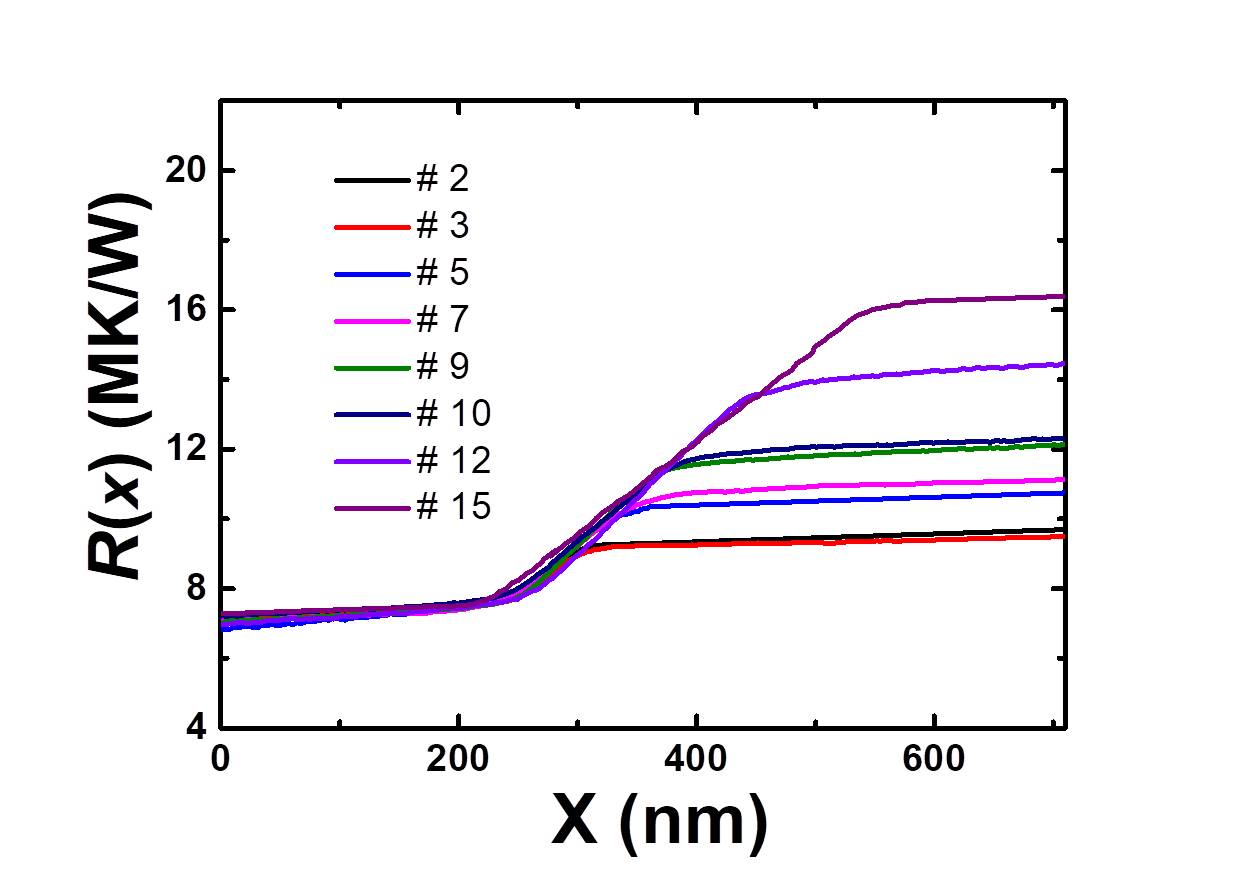


**Supplementary Figure 5 | Length-dependent cumulative thermal resistance of silicon nanowires across the amorphous regions.** The samples are #2, #3, #5, #7, #9, #10, #12 and #15, and the rest is shown in Figure 2 (b) in the main text. The slope of amorphous regions overlaps with each other, even for different nanowires with different amorphous lengths, implying that the amorphous regions with various lengths keep constant thermal conductivity, which is calculated as 1.9±0.25 W/m.K. The thermal conductivity of crystalline silicon parts is 50.2±2.3 W/m.K, obtaining by linearly fitting the curves of crystalline regions and taking average of different samples.

**
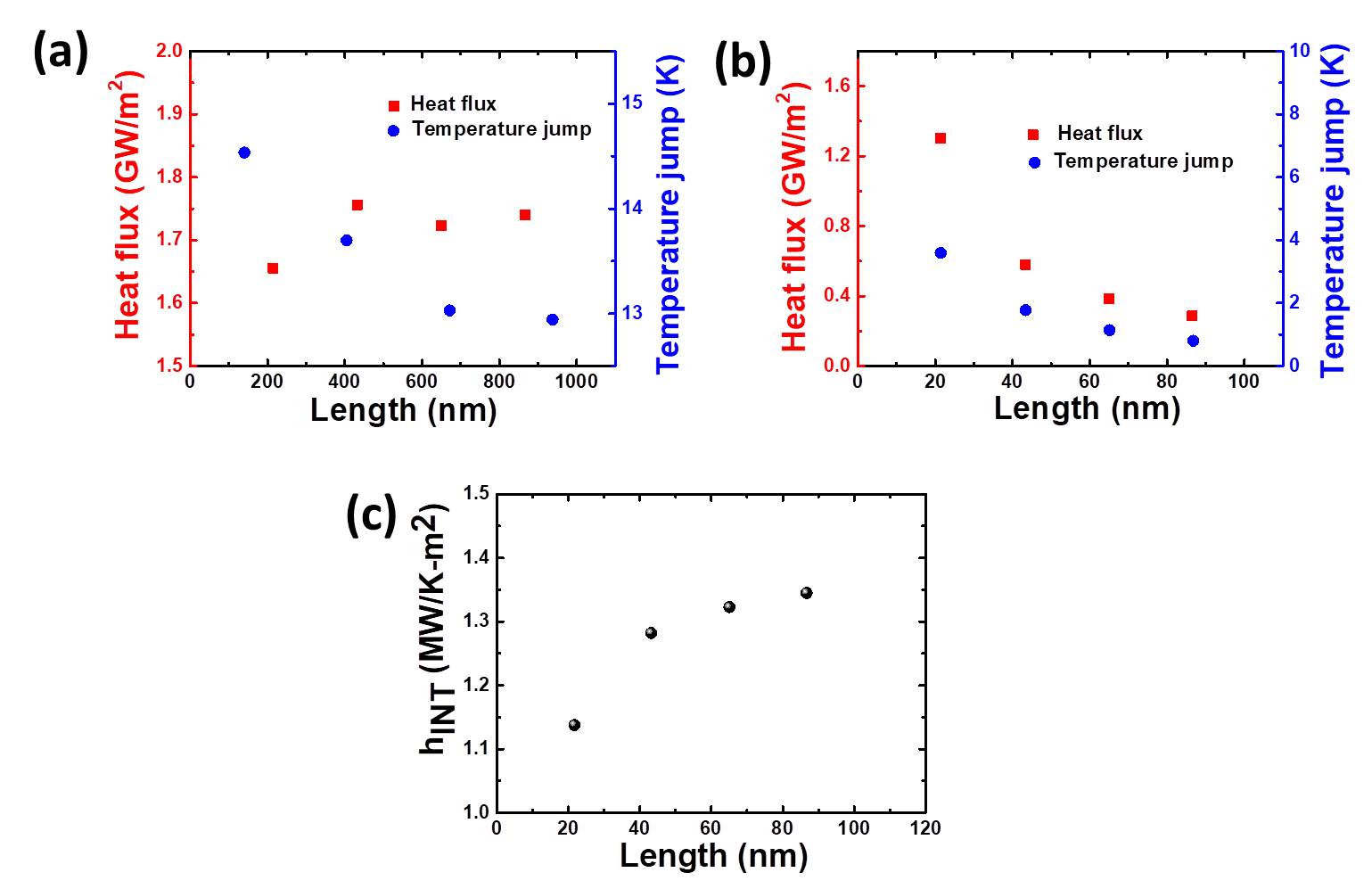
**

**Supplementary Figure 6 | Molecular dynamics (MD) calculation of interface thermal conductance.** (a) and (b) are length-dependent heat flux and temperature jump for crystalline/crystalline and crystalline/amorphous interface, respectively. (c) is length-dependent interface thermal conductance for crystalline/crystalline interface. The length-dependent interface thermal conductance for crystalline/amorphous interface is shown in Figure 4 in the main text.


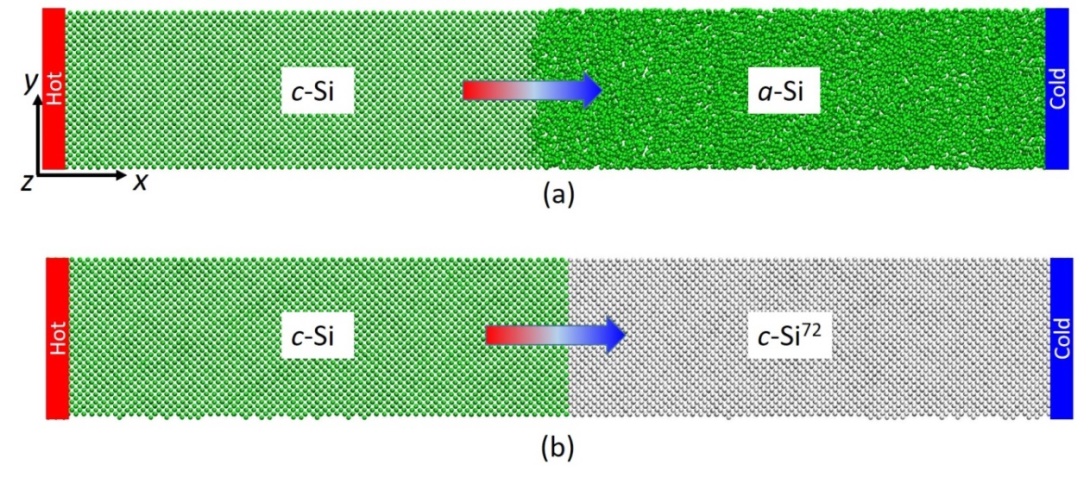


**Supplementary Figure 7 | MD simulation of interface thermal conductance** (a) c-Si/a-Si structure and (b) c-Si/c-Si^72^ structure with hot and cold baths at the ends.


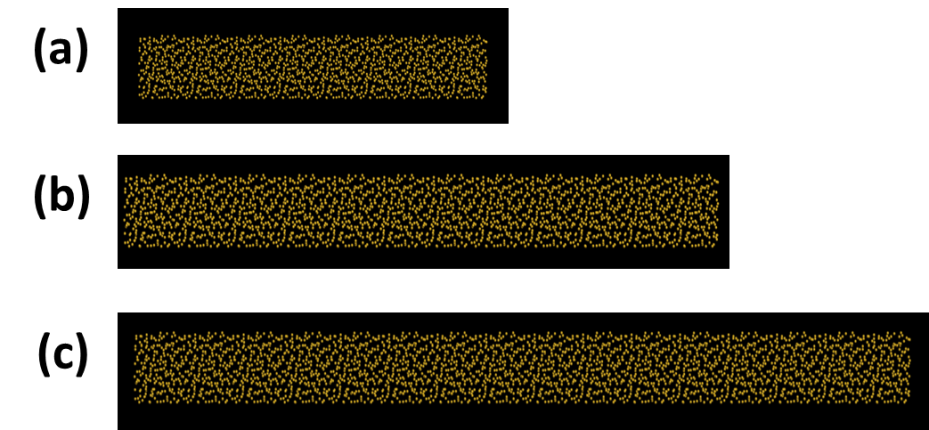


**Supplementary Figure 8 | Molecular dynamics (MD) calculation for density of states (DOS) of amorphous silicon nanowires.** (a), (b) and (c) is amorphous silicon nanowires with lengths of 12.83 nm, 19.25 nm and 25.66 nm, respectively.

**Supplementary Note 1 | Increase ratio of thermal resistance of measured silicon nanowires.**

The measurement of *α_0_*, that is the ratio of temperature rise between right membrane and left membrane, would give information for the thermal resistance of the whole measured silicon nanowire. *α_0_* was measured before and after helium ion irradiation for all the fifteen METS devices. Considering *R_T_*=(*α_0_*-1) *R_b_*, we calculate the increase ratio of thermal resistance of some measured nanowires and the increase ratio is defined as Ratio = (*R_T, irradiation_* - *R_T,non-irradiation_*)/*R_T,non-irradiation_*, which is actually (*α_0,irradiation_*-*α_0,non-irradiation_*)/*a_0,non-irradiation_*, where *R_T_,* *_irradiation_* and  *R_T,non-irradiation_* are for the total thermal resistance of silicon nanowire with and without irradiation, and *α_0,irradiation_* and *α_0,non-irradiation_* are for *a_0_* with and without irradiation, respectively. It can be seen that the increase ratio is linearly dependent on the irradiation length (*L*) as well, as shown in Supporting Information Figure S5. Considering constant *R_b_* and contact thermal resistance between nanowires and left (right) Pt loops before and after helium ion irradiation, the total thermal resistance increase, coming from the helium ion irradiation, would lead to the increase in *α_0_* directly and thus the final increase ratio of *α_0_*.

**Supplementary Note 2 | Molecular dynamics (MD) simulation for interface thermal conductance of crystalline/amorphous silicon**

In this work, two interface systems are considered. The first one is crystalline silicon/amorphous silicon (c-Si/a-Si) system; the second one is crystalline silicon/ crystalline isotopic silicon (c-Si/c-Si72). It should be noted that, in order to highlight the mass effect and reduce the lattice mismatch at the interface, here we assume the atomic mass of isotopic silicon is 72.64, which is equal to germanium atomic mass.

The *c*-Si/*a*-Si system is shown in Supplementary Figure S7 (a). The longitudinal (*x*) direction is set along the [100] direction. The *a*-Si region is generated by heating crystalline silicon to their melting point and then quenching them to room temperature. In order to precisely control the size of crystalline silicon region, the initial configuration is created by fixing the atoms in the crystalline part, and melting the other part at a temperature of 2400 K. Then the system is quenched to room temperature with a cooling rate of 10^3^ K/ns. Finally, the whole system is annealed at 600 K for 800 ps. Periodic boundary condition is applied in *y* and *z* directions to mimic the infinite width of the heterostructures. The *c*-Si/*c*-Si^72^ system is shown in Supplementary Figure S7 (b) with its longitudinal direction along the [100] direction.

In all molecular dynamics (MD) simulations performed herein, interatomic forces were described using Stillinger-Weber potential^1^, and simulations were carried out with the large-scale atomic/molecular massively parallel simulator (LAMMPS)^2^ with a time step of 1 fs throughout. The lattice constant of crystal silicon is set *a* = 5.43 Å. The velocity Verlet algorithm is employed to integrate Newton’s equations of atom motion numerically. When the structure was generated following the approach introduced above, the system was relaxed with *NPT* (constant pressure and temperature) ensemble. After the relaxation, the system was then equilibrated at a constant temperature of *T* = 300 K for 500 ps using Nosé-Hoover temperature thermostat^3^. After the constant temperature relaxation, we continued to relax the system with *NVE* (constant volume and no thermostat) ensemble for 1 ns. During this stage, the total energy and temperature of the system were monitored. We found that the total energy was conserved and the temperature of the entire system remained constant with fluctuations around 300 K, which means that the system has reached equilibrium. The same methodology has also been used to generate crystalline-core amorphous-shell silicon nanowires^4, 5^.

In the equilibration stage, we computed the interfacial thermal conductance (ITC) of the system with non-equilibrium MD (NEMD) method. To establish a temperature gradient along the longitudinal direction, the atoms close to the two ends of the system were placed into hot and cold Nosé-Hoover reservoirs with temperatures set to be T_H_ = 310 K and T_C_ = 290 K, respectively, as shown in Supplementary Figure S7 (a) and (b). The simulations are then performed long enough (10 ns) to allow the system to reach the non-equilibrium steady state where the temperature gradient is well established, and the heat flux going through the system is time-independent. In NEMD, the value of ITC can be calculated by $G=\frac{J}{\Delta T}$, where *G* is the ITC, *ΔT* is the temperature jump at the interface, and *J* is the heat flux across the interface, which was calculated by using^2^:

(S1)

where, $\varepsilon_{i}$ and $\mathbf{v}_{i}$are the energy and velocity associated with atom *i*, respectively. Vector ***r****_ij_*denotes the interatomic distance between atoms *i* and *j*, and ***F****_ij_* and ***F****_ijk_* denote the two-body and three-body force, respectively. *V* is the volume of the studied system. After the system reached the non-equilibrium steady state, a time averaging of temperature and heat flux was performed for additional 30 ns. To avoid the nonphysical effect of heat source and heat sink at the two ends, the regions (2-unit cell long) close to both heat reservoirs are excluded for the calculation of heat flux.

We carried out a series of NEMD simulations for *c*-Si/*a*-Si and *c*-Si/*c*-Si^72^ systems. In the simulations, the thickness of *c*-Si, *D_c_*, in all these systems are same, *D_c_* = 40*a* (21.72 nm), but different thickness of *a*-Si (*c*-Si^72^), *Da* (*D*^72^), are considered, where *Da* (*D*^72^) = 40*a*, 80*a*, 120*a*, 160*a*. All the systems have the same cross-sectional size, 5*a* × 5*a* with periodic boundary condition in *y* and *z* directions to mimic the infinite width of the systems. Figure S6 (c) shows the ITC of *c*-Si/*c*-Si^72^ system as a function of the thickness of *c*-Si^72^. It is found that the ITC increases with the increasing thickness of *c*-Si^72^. This is due to more long-wavelength phonon modes existing in the thicker *c*-Si^72^, which contribute additional heat carriers across the interface.

**Supplementary Note 3 | Molecular dynamics (MD) simulation for density of states (DOS) of amorphous silicon nanowires**

Classical molecular dynamics simulations are carried out using the LAMMPS package^2^ to obtain the phonon densities of states (PDOS) of amorphous Si nanowires with different length 12.83 nm，19.25 nm and 25.66 nm. The Stillinger-Weber potential^1^ is used in this study to represent the interactions between the atoms, and the velocity Verlet algorithm^6^ is employed to numerically integrate the motion equations with an integration time step of 0.1 fs. The PDOS are computed from the Fourier transform of the velocity autocorrelation function (VACF)^7^,

$g(\omega)=\frac{1}{\sqrt{2\pi}}\int_{0}^{\infty} e^{i\omega t}\sum_{j=1}^{N} \frac{\left\langle\upsilon_{j}(t)\upsilon_{j}(0)\right\rangle}{\left\langle\upsilon_{j}(0)^{2}\right\rangle}dt$, (S2)

where $\omega$ is the phonon frequency, the $\upsilon_{j}(0)$ is the average velocity vector of the $j$th particle at initial time, and the $\upsilon_{j}(0)$ is the average velocity vector of the $j$th particle at time $t$.

**Reference**

1. F. H. Stillinger, T. A. Weber, *Phys. Rev. B* 1985, **31**, 5262.

2. S. Plimpton, *J. Comput. Phys.* 1995, **117**, 1-19.

3. S. Nosé, *J. Chem. Phys.* 1984, **81**, 511-519.

4. X. Liu, G. Zhang, Q. Pei, Y. Zhang, *Sci. China Tech. Sci.* 2014, **57**, 699-705.

5. X. Liu, G. Zhang, Q.-X. Pei, Y.-W. Zhang, *Mater. Today. Proc.* 2016, **3**, 2759-2765.

6. M. Tuckerman, B. J. Berne, G. J. Martyna, *J. Chem. Phys.* 1992, **97**, 1990-2001.

7. J. Dickey, A. Paskin, *Phys. Rev.* 1969, **188**, 1407.
